# Student Performance in Bitewing Caries Detection: Artificial Intelligence Versus Alternative E-learning

**DOI:** 10.1016/j.identj.2026.109567

**Published:** 2026-04-30

**Authors:** Valéria Nagyová, Dominik Blaňár, Jan Kybic, Falk Schwendicke, Antonín Tichý

**Affiliations:** a Institute of Dental Medicine, First Faculty of Medicine, Charles University and General University Hospital, Prague, Czech Republic; b Faculty of Electrical Engineering, Czech Technical University, Prague, Czech Republic; c Clinic of Conservative Dentistry, Periodontology and Digital Dentistry, LMU University Hospital, Munich, Germany

**Keywords:** Caries, Bitewing, Artificial intelligence, Convolutional neural network, Dental students

## Abstract

**Introduction and Aims:**

This study compared 3 methods for teaching caries detection in bitewings: a prerecorded lecture, a preannotated dataset, and an artificial intelligence (AI)–based web application.

**Methods:**

Fifty-two dental students annotated carious lesions in 50 bitewings using minimum bounding boxes. After initial annotations, students were divided into 3 groups according to the training method: the Lecture Group (n = 16) received a prerecorded lecture on caries detection in bitewings, the Dataset Group (n = 17) had access to 50 bitewings annotated by a dentist, and the AI Group (n = 19) used an AI-based web application. After training, all students annotated caries in 50 previously unseen bitewings. Student annotations before and after training were compared to a reference standard of 3 experienced dentists. The evaluation was stratified according to the training method and stage of studies: preclinical (n = 16), junior clinical (n = 15), and senior clinical (n = 21).

**Results:**

All training methods significantly improved the mean number of errors, intersection over union of matching annotations, and accuracy. Sensitivity increased significantly in the Dataset Group (from 0.62 ± 0.14 to 0.78 ± 0.08) and the AI Group (from 0.68 ± 0.15 to 0.73 ± 0.12), as opposed to the Lecture Group, where a significant increase in specificity was observed (from 0.94 ± 0.09 to 0.96 ± 0.05). The stage of studies impacted the results; the extent of improvement decreased with increasing clinical experience.

**Conclusion:**

While the 3 training methods varied in their impact on the confusion matrix components, they yielded comparable overall improvements. The AI-based web application could serve as an educational tool for caries detection in bitewings, especially for dental students with limited clinical experience.

**Clinical Relevance:**

This study shows that learning bitewing caries detection with an AI tool yields improvements comparable to other tested e-learning methods. Evaluating and comparing established e-learning and AI teaching methods is key to optimising AI-assisted education for better learning outcomes in dental training.

## Introduction

Intraoral radiographs, particularly bitewings, are frequently used as an adjunct method to visual-tactile detection of caries.^1^ While visual-tactile examination is particularly useful for detecting cavitated occlusal or cervical lesions, bitewings are more suitable for the diagnosis of proximal caries lesions.^2^ However, radiographic caries detection has limitations, including the risk of ionising radiation and reported low sensitivity for initial lesions,^2^ and the subjective nature of interpretation by dental professionals of varying experience, who may lack the specialised training or expert supervision of radiologists. The low interobserver agreement has been reported by several studies,^2^^,^^3^ despite the fact that detecting caries using radiography is one of the major competencies a dentist must develop prior to graduation.^4^

Traditional curricula teach bitewing interpretation through a combination of lectures (traditional face-to-face learning or e-learning methods), preclinical exercises using radiographic sets, and clinical supervision during patient care provided by experienced instructors. Even though traditional learning methods have proven effective in both cariology^5^, ^6^, ^7^ and oral radiology education,^8^^,^^9^ clinical training remains particularly susceptible to the interobserver variability, which can hinder consistent skill development, and is further constrained by the time-consuming nature of individualised supervision, as students often spend considerable time waiting for confirmation and feedback.^10^ Given that students’ acceptance of learning is shaped by the type of their training^11^ and the feedback they receive,^12^ targeted innovations in these areas hold potential to strengthen learning outcomes.

To address these challenges, curricula have increasingly adopted multimodal approaches and standardised scoring systems for lesion staging.^13^ Moreover, augmented reality simulators with haptic technology have been integrated as adjuncts to traditional preclinical teaching,^14^ but applications of virtual and augmented reality in caries detection training remain limited.^15^ Recent attention has shifted toward artificial intelligence (AI)–based models that offer automated caries detection.^16^, ^17^, ^18^, ^19^, ^20^, ^21^ In studies that included a comparison with human annotators,^3^^,^^16^^,^^18^^,^^22^^,^^23^ the AI models usually performed similarly to experienced dentists or dentomaxillofacial radiologists and outperformed the less experienced ones.

From a clinical perspective, it is even more relevant how the use of AI-based models affects the performance of dentists, as this shapes the training and learning outcomes of the students they supervise. This has been addressed by only a handful of studies, which indicated that AI-based tools may increase dentists’ sensitivity in detecting enamel lesions^24^^,^^25^ and alter the way they inspect the AI-labelled image.^26^ In addition, a clinical trial by Mertens et al^25^ identified 2 notable challenges: first, even though most participants showed improvements while using AI, its effect was very variable; second, the use of AI led to an increase in both invasive and noninvasive treatment decisions. Several studies also found that many clinicians and students believe AI will revolutionise dentistry or enhance diagnostics.^27^, ^28^, ^29^, ^30^

Nevertheless, the adoption of AI in dental education remains limited despite growing enthusiasm.^29^, ^30^, ^31^, ^32^, ^33^ Recent studies reported promising results of training students using AI-based diagnostic models for radiographic analysis,^34^, ^35^, ^36^, ^37^ but these studies did not consider students’ experience or include direct comparisons with established teaching methods. Such comparisons are essential to determine whether AI truly enhances students’ caries detection performance beyond established approaches, thereby providing a basis for the evidence-based integration of AI into dental curricula while maintaining educational quality and patient safety.

In this study, students in different stages of their studies received training in caries detection using 1 of 3 training methods, including a prerecorded lecture, a dataset of bitewings with annotated caries, and an AI-based web application for caries detection. Students’ performance before and after training was compared with that of experienced dentists, and the null hypotheses were that performance would be influenced neither by the training method nor by the stage of studies.

## Materials and methods

### Data

A publicly available dataset of 100 bitewings^38^ was used in this study. The bitewings were acquired using 4 different intraoral x-ray units with sensor physical dimensions ranging from 31 × 41 mm to 27 × 54 mm. For this dataset, only radiographs depicting permanent dentition and including a minimum of 4 teeth were selected.

Bitewings presenting extensive artifacts or large overlaps of adjacent proximal surfaces were not included in the dataset, but the inclusion was not limited by the presence or size of caries and restorations.

The 100 bitewings were randomly divided into 2 datasets of 50 images each: the initial dataset ($D_{0}$) and the testing dataset ($D_{1}$). Both datasets were reviewed by a dentist with 5 years of experience (V.N.), who ensured an even distribution of both healthy and carious tooth surfaces in each dataset, resulting in a total of 1008 surfaces in dataset $D_{0}$ and 993 surfaces in dataset $D_{1}$.

Prior to the start of this study, both datasets were annotated by 3 external experienced dentists, each with more than 15 years of professional experience (experts),^3^ who were not participating in other tasks. Neither the experts nor the participating students had access to any clinical information about the patients. The study was therefore conducted purely as a radiologic evaluation based solely on the interpretation of bitewings.

### Participants

This study employed a convenience sampling approach. The group size was determined by feasibility, as the number of available volunteers was limited. All students who completed the study were included.

A total of 60 dental students from the First Faculty of Medicine, Charles University, Prague, Czech Republic, were initially enrolled in the study. Of these, 8 students discontinued participation and were therefore excluded from the final analysis, resulting in a total of 52 participants who completed the study.

The students were divided into 3 groups according to the stage of their academic studies:

• Preclinical students (first and second academic year, n = 16). During this period, student-patient contact is minimal, and students receive basic instruction in caries detection and interpretation of bitewings through the courses of preclinical dentistry and cariology.
• Junior clinical students (third academic year, n = 15). This period marks the beginning of more intensive patient contact. Clinical training is delivered separately within individual dental fields, and knowledge is not yet integrated across fields as it is in the senior clinical group. Students receive further instruction on bitewing caries detection in courses on preventive and restorative dentistry.
• Senior clinical students (fourth and fifth academic year, n = 21). From the middle of the fourth and throughout the fifth academic year, students attend daily clinical training, providing comprehensive care to patients presenting with conditions spanning all dental fields. Additionally, at the end of the fourth year, students are required to complete a mandatory summer clerkship in dentistry.

**Fig. 1:**
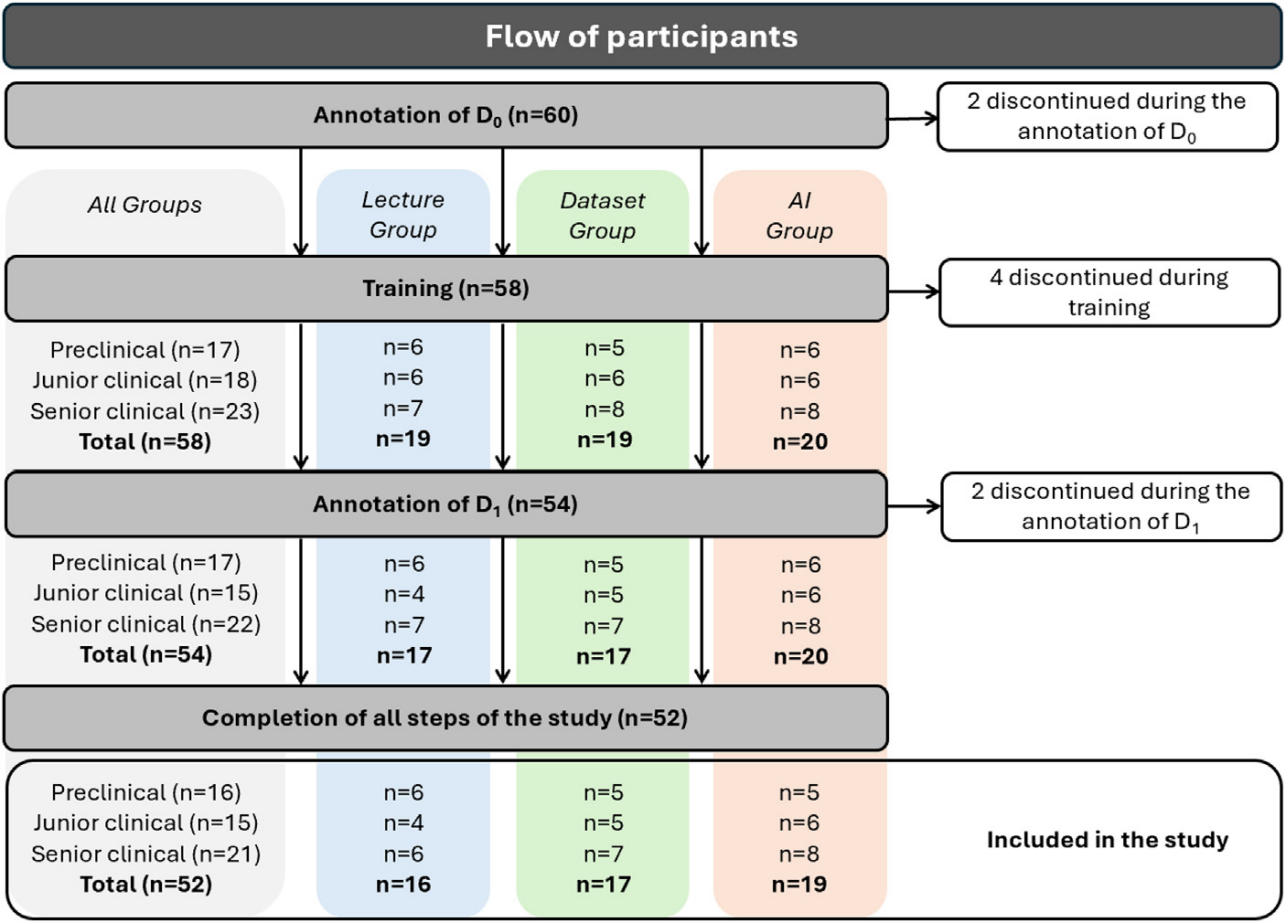
Flowchart illustrating the enrolment and dropout of students in each training group and academic stage.

The study was conducted at the end of the academic year, after the students had completed the curriculum for that year. See Figure 1 for details about enrolment, dropout, and group distribution of participating students.

### Study design

All students were instructed how to use the Label Studio annotation software^39^ for caries detection with minimum bounding boxes and annotated the dataset $D_{0}$. Following the initial annotations, students were randomly assigned to 3 groups according to the employed training method. The Lecture Group (n = 16) was given a brief online lecture prerecorded by a dentist with 5 years of experience (V.N.), explaining the basic principles of caries detection on bitewings, along with the most common mistakes demonstrated on 15 images from dataset $D_{0}$. The lecture lasted approximately 6 minutes and was delivered either in Czech or English. The Dataset Group (n = 17) was trained using dataset $D_{0}$ with carious lesions annotated by the same dentist (V.N.), simulating case-based learning led by a junior instructor, although the students reviewed the dentist’s annotations without additional guidance. When evaluated against the reference standard (see chapter Reference standard for details), the annotator achieved 88% sensitivity and 97% specificity (see Table 1 for the complete set of performance metrics for this dataset). The AI Group (n = 19) trained using an AI-based web application with the 50 images from dataset $D_{0}$ available for inference. The web application was based on the best-performing model from a previous project (ie, an ensemble of 4 different object detection architectures: RetinaNet-SwinT, Faster R-CNN-ResNet50, YOLOv5-M, and RetinaNet-R101).^19^ To develop the model, a dataset of 3989 bitewings was compiled and annotated with 7257 labels by a dentist specialised in cariology (A.T., then with 5 years of experience). The key limitation of the model is that all annotations were performed by a single expert.^19^ The web application used for training of the AI Group generated bounding boxes indicating the presence of carious lesions with 78% sensitivity and 99% specificity (see Table 1 for the complete set of performance metrics), and it featured a slider for adjusting the model’s confidence threshold, which influences the sensitivity and precision of the model. Students in this group received a dentist-prepared (V.N.) user manual containing illustrated, step-by-step instructions for using the application, including guidance on running automated analysis on the dataset $D_{0}$, uploading additional bitewings (optional), and adjusting the confidence threshold. After the training, all students annotated caries in the dataset $D_{1}$.

**Table 1:** Performance of the dentist-annotator (V.N.) and the AI application on the initial dataset ($D_{0}$) compared with the reference standard.

	Errors	IoU	Sensitivity	Specificity	Accuracy	Precision	F1 score	TP	FN	FP	TN
Annotator for the Dataset Group	40	0.54	0.88	0.97	0.96	0.83	0.85	116	16	24	801
AI application for the AI Group	39	0.49	0.78	0.99	0.96	0.91	0.84	103	10	29	815

Abbreviations: AI, artificial intelligence; IoU, intersection over union; FN, false negative; FP, false positive; TN, true negative; TP, true positive.

The initial dataset ($D_{0}$) for the Dataset Group was annotated by a dentist with 5 years of clinical experience (V.N.). The AI Group used an AI application that generated bounding boxes around carious lesions. The number of errors is the sum of FPs and FNs.

### Reference standard

To evaluate students’ annotations, a reference set of annotations was established by 3 external experts. The expert annotations were considered to correspond to the same lesion if the centroid of 1 annotation lay inside the other or vice versa. The annotations were matched automatically using an algorithm, and annotations corresponding to the same lesion were subsequently combined (see Tichý et al^3^ for details). Each lesion was then assigned to the majority set S_234_ (from now on referenced as S) if it was marked by at least 2 of the 3 experts and its corresponding minority set S′_23__4_ (from now on referenced as S′) if it was marked by only 1 expert. Lesions marked in set S were considered the ground truth, and the lesions in set S′ were considered tentative, which were counted as neither true-positive nor false-positive detections.

The automatic matching and transfer of annotations to sets S and S′ were evaluated by a dentist (V.N.), who identified 11 instances of incorrect matching in the total of 7 images in both datasets $D_{0}$ and $D_{1}$, particularly in images where lesions were located in close proximity, such as adjacent proximal surfaces. In these instances, the transfer to the reference standard was manually adjusted.

Overall, the initial dataset $D_{0}$ consisted of 825 healthy surfaces, 132 carious lesions in set S, and 51 carious lesions in set S′, whereas the testing dataset $D_{1}$ included 815 healthy surfaces, 125 carious lesions in set S, and 53 carious lesions in set S′. The prevalence of caries in our dataset was estimated by calculating the ratio of annotated caries cases in sets S and S′ to the total number of surfaces. Specifically, the prevalence of caries per tooth surface was approximately 13.1% to 18.2% in $D_{0}$ and 12.6% to 17.9% in $D_{1}$.

### Comparison with the reference standard

Bounding boxes of each student were compared against both sets S and S′ of the reference standard. The student’s annotation was considered to match the annotation of the reference standard (ie, the bounding boxes correspond to the same lesion), if the centroid of one annotation lay inside the other or vice versa. Students’ annotations that matched the boxes in the majority set S were counted as true positives (TPs). Students’ annotations that matched the boxes in the minority set S′ were counted as neither TP nor false positive (FP). Students’ annotations that did not match annotations from either the majority set S or minority set S′ were considered FP. Finally, annotations in the majority set S that were left unmatched by the students’ annotations were counted as false negatives (FNs).

Note that in the detection formulation, there is no clear definition of true negatives (TNs). The number of TN annotations was approximated based on the assumption that the maximum number of carious lesions corresponds to the total number of tooth surfaces, with each surface potentially affected by only 1 lesion. This assumption represents an approximation, as carious lesions may span multiple surfaces, and in rare cases, multiple lesions may occur on a single surface. The number of TNs was calculated by subtracting from the total number of tooth surfaces the sum of TPs, FPs, FNs, and tentative annotations in the minority set S′ (Eq. (1)).

(1) $$\mathrm{TN}=N_{surfaces}-\sum \left(\mathrm{TP}+\mathrm{FP}+\mathrm{FN}+N_{tentative}\right)$$

The number of errors was calculated as the sum of FPs and FNs (ie, the sum of unmatched student annotations and unmatched boxes in majority set S). The intersection over union (IoU) was calculated for each pair of corresponding student annotations and annotations in the majority set S that were matching. The mean values were calculated per annotator over all images.

For a detailed comparison with the reference standard, including formulas and algorithms, see Appendix A.

**Fig. 2:**
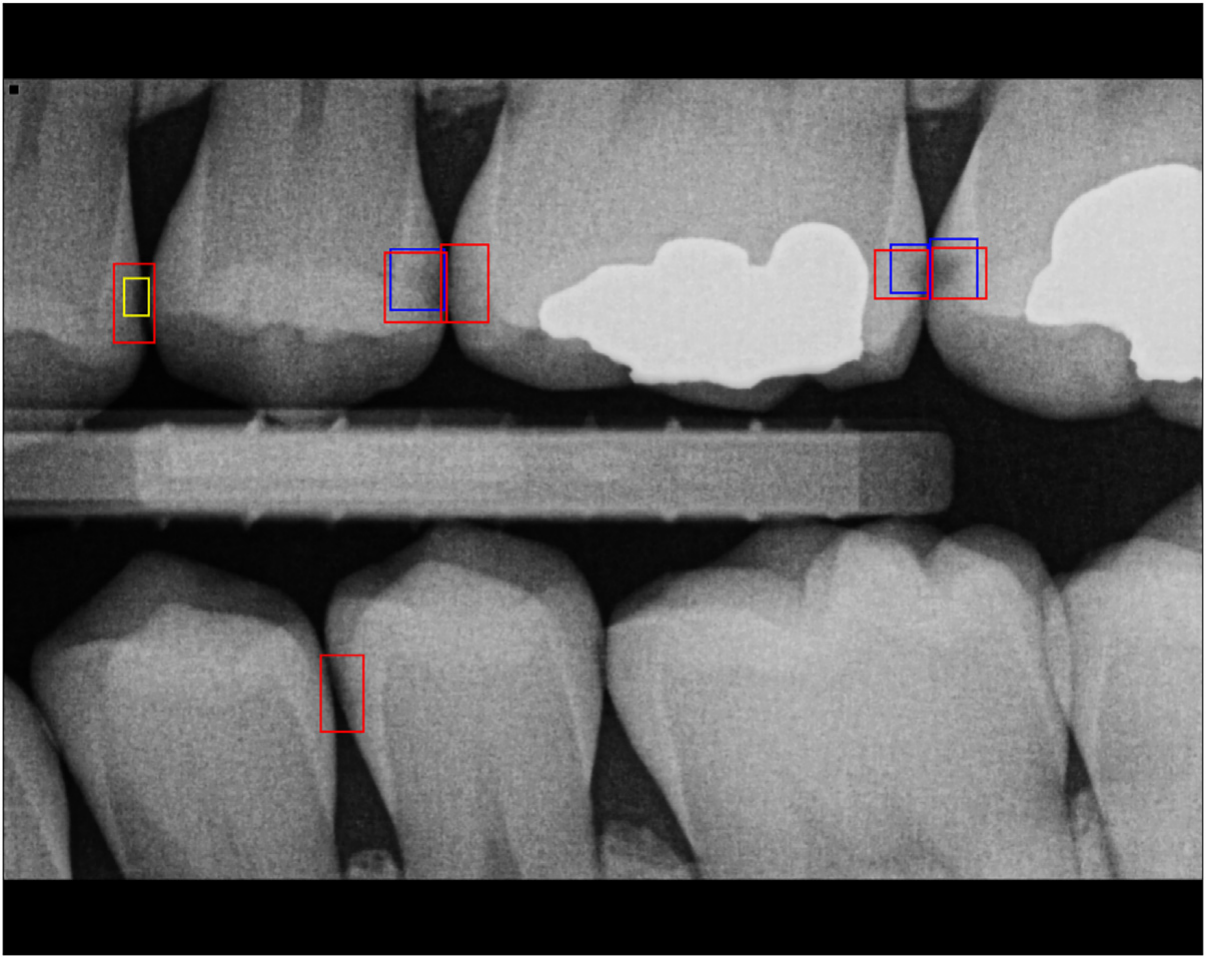
Sample bitewing radiograph with annotations of a student annotator (red), reference standard majority set S (blue), and minority set S′ (yellow).

Figure 2 illustrates an example of an evaluated image. Annotations from the reference standard set S are displayed in blue, while those from set S′ are shown in yellow. The red annotations represent labels provided by a selected student. Based on the comparison with the reference standard, the image contains 3 TP annotations (overlapping red and blue annotations); 1 tentative annotation (overlapping red and yellow annotation), which is counted as neither TP nor FP detection; and 2 FP annotations (red annotations without any match). Consequently, the total error count for this image amounts to 2.

### Statistical testing

We used object detection metrics such as IoU of the matching annotations and the number of errors, similar to our previous study.^3^ The number of errors reflects the correctness of lesion detection, while IoU measures how closely the students’ annotations aligned with the reference standard. However, these metrics may have limitations, as the reference standard represents a synthesis of annotations from multiple experts, each of whom may have marked the lesions differently.

For each annotator, cumulative values of TP, FP, FN, and TN annotations across all images were used to compute performance metrics, including sensitivity, specificity, accuracy, precision, and F1 score, which are often used in dental computer vision studies.^40^ Sensitivity was defined a priori as the primary outcome metric, while all remaining metrics were considered exploratory. The performance metrics were summarised across annotators within each group. Mean values and standard deviations were reported, along with median values and interquartile ranges.

For all analyses, the significance level was set at .05. To check the assumption of normality, the Shapiro-Wilk test was used, which revealed that the data for more than half of the metrics were not normally distributed. Based on these results, we conducted permutation tests to determine the statistical significance of the observed improvements, because they do not rely on distributional assumptions. To compare the effectiveness of different training methods and academic stages, the Kruskal-Wallis H-test was applied to the improvement of each group from baseline (ie, the difference between pre- and posttraining metrics for each student). Dunn’s test with Bonferroni correction was used as a post hoc analysis to identify specific group pairs with significant differences in their medians.

## Results

The analysis of all 52 students combined showed statistically significant improvements (*P* < .05) in all diagnostic metrics when comparing their results before and after training, as shown in Table 2.

**Table 2:** Pre- and posttraining results of all 52 students.

Characteristic	Errors	IoU	Sensitivity	Specificity	Accuracy	Precision	F1 score
All students pretraining	85.4 ± 51.2	0.45 ± 0.09	0.68 ± 0.14	0.95 ± 0.06	0.91 ± 0.05	0.73 ± 0.15	0.69 ± 0.11
All students posttraining	63.2 ± 27.2	0.50 ± 0.09	0.75 ± 0.11	0.96 ± 0.04	0.93 ± 0.03	0.78 ± 0.13	0.75 ± 0.08
*P* value	**<.0001**	**<.0001**	**.0011**	**.0411**	**.0001**	**.0186**	**<.0001**

Abbreviation: IoU, intersection over union.

Values are presented as mean ± standard deviation. The number of errors is the sum of false positives and false negatives. The bold *P* values indicate statistically significant improvement between pre- and posttraining results according to the permutation test (*P* < .05).

Subsequent analyses were conducted to assess the differences between the compared training methods and stages of academic study. The Kruskal-Wallis H-test results, summarised in Table 3, revealed significant differences in the improvement of the primary outcome metric (sensitivity), along with the F1 score, between training methods. The post hoc analysis and comparison with the original results then showed that training with the annotated dataset led to a significantly greater improvement in detection performance and reliable positive predictions, compared to the lecture-based training (post hoc test results for difference between improvement in the Lecture Group and the Dataset Group in sensitivity and F1 score: *P* = .0021 and *P* = .0347, respectively). No significant differences were found between the AI Group, which underwent training on bitewings labelled with AI-generated bounding boxes, and the other groups. Additionally, no significant differences were found between improvements across stages of academic study.

**Table 3:** Kruskal-Wallis test of improvements between pre- and posttraining scores across groups classified by training method and academic stage (*P* values, significance threshold = .05).

Characteristic	Errors	IoU	Sensitivity	Specificity	Accuracy	Precision	F1 score
Training method	.3438	.0945	**.0024**	.4983	.3230	.7256	**.0407**
Academic stage	.1191	.3152	.4361	.1332	.1180	.1928	.0772

Abbreviation: IoU, intersection over union.

The bold *P* values indicate statistically significant differences. Training method refers to the Lecture Group, the Dataset Group, and the AI Group. Academic stage refers to groups of preclinical students, junior clinical students, and senior clinical students. The number of errors is the sum of false positives and false negatives.

### Training method

All groups significantly improved in caries detection, indicated by a decline in the number of errors and an increase in the IoU of the matching annotations. Table 4 summarises these results across all metrics for each training method, presenting the mean values and standard deviations. The AI Group showed the greatest reduction in the average number of errors after training, yet its median improvement was comparable to the Lecture Group and smaller than that of the Dataset Group (see Table B.7 in Appendix B for the exact median values and interquartile range [IQR] for each group). This suggests that while a few individuals in the AI Group improved significantly, overall improvement across the group was less consistent.

**Table 4:** Pre- and posttraining results of the Lecture Group, the Dataset Group, and the AI Group.

Characteristic	Errors	IoU	Sensitivity	Specificity	Accuracy	Precision	F1 score
Lecture Group pretraining	83.6 ± 68.4	0.48 ± 0.09	0.75 ± 0.12	0.94 ± 0.09	0.91 ± 0.07	0.74 ± 0.17	0.72 ± 0.11
Lecture Group posttraining	65.3 ± 35.5	0.53 ± 0.10	0.73 ± 0.12	0.96 ± 0.05	0.93 ± 0.04	0.79 ± 0.15	0.74 ± 0.09
*P* value	**.0049**	**.0003**	.7309	**.0226**	**.0092**	**.0321**	.1358
Dataset Group pretraining	86.2 ± 34.3	0.43 ± 0.08	0.62 ± 0.14	0.96 ± 0.04	0.91 ± 0.04	0.74 ± 0.14	0.66 ± 0.09
Dataset Group posttraining	63.8 ± 26.7	0.50 ± 0.06	0.78 ± 0.08	0.96 ± 0.04	0.93 ± 0.03	0.76 ± 0.12	0.76 ± 0.07
*P* value	**.0302**	**.0006**	**.0001**	.5167	**.0381**	.3757	**.0008**
AI Group pretraining	86.2 ± 49.9	0.44 ± 0.09	0.68 ± 0.15	0.95 ± 0.06	0.91 ± 0.05	0.72 ± 0.15	0.68 ± 0.12
AI Group posttraining	60.8 ± 19.9	0.47 ± 0.09	0.73 ± 0.12	0.97 ± 0.02	0.94 ± 0.02	0.79 ± 0.11	0.75 ± 0.08
*P* value	**.0037**	**.0481**	**.0450**	.0647	**.0053**	**.0314**	**.0032**

Abbreviation: IoU, intersection over union.

Values are presented as mean ± standard deviation. The number of errors is the sum of false positives and false negatives. The bold *P* values indicate statistically significant improvement between pre- and posttraining results according to the permutation test (*P* < .05).

**Fig. 3:**
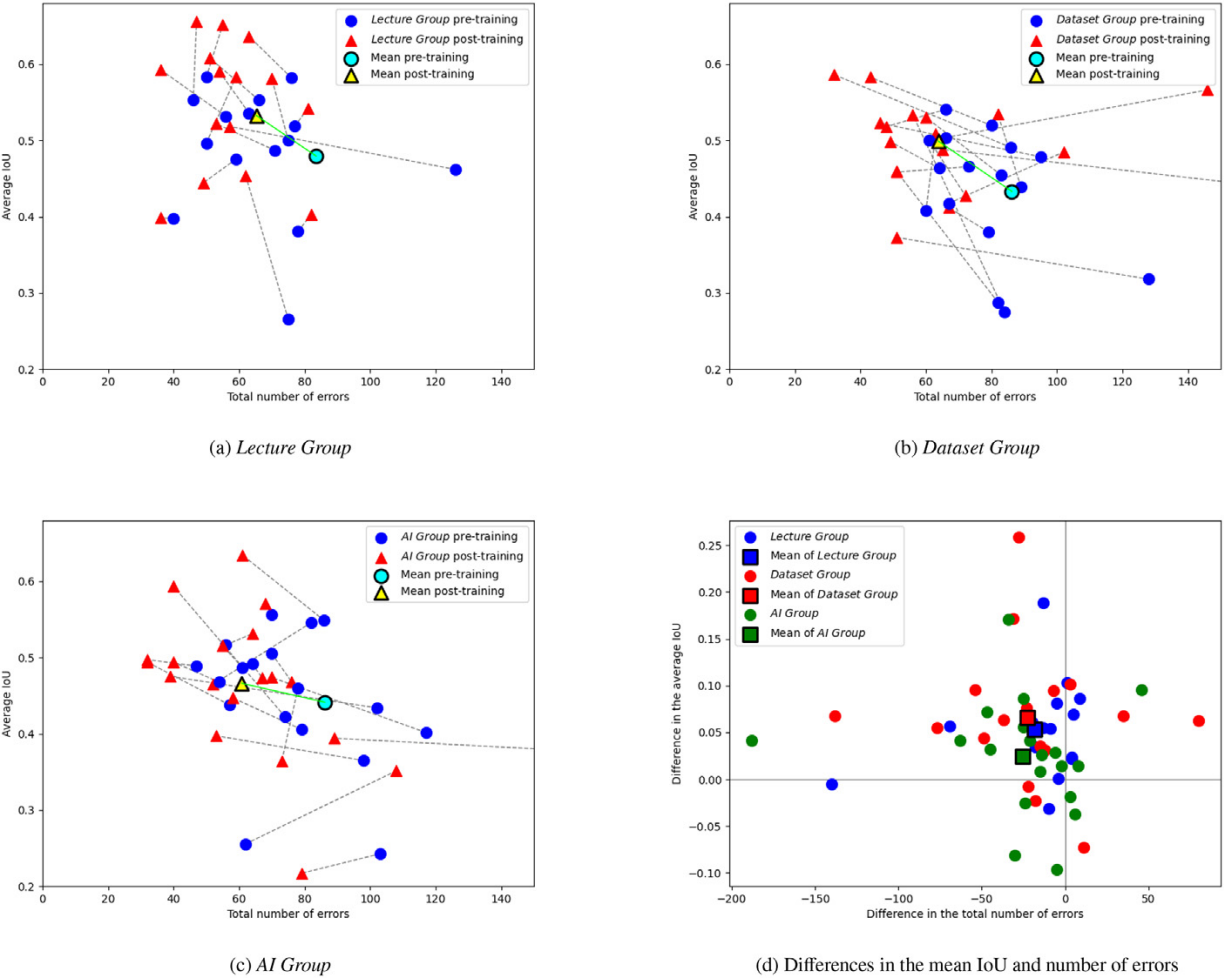
Mean IoU and total number of errors for students in the Lecture Group (a), the Dataset Group (b), and the AI Group (c) and their differences for students of all groups (d). Lines connect the pretraining and posttraining results of each student. An ideal result would be in the top left. For improved readability of the figure, the total number of errors displayed on the x-axis was capped at 150. Three outliers were affected by this cap: 1 in the Lecture Group (improvement from 329 to 189 errors), 1 in the Dataset Group (improvement from 203 to 65 errors), and 1 in the AI Group (improvement from 277 to 89 errors). IoU, intersection over union.

The results of individual students in terms of the number of errors and IoU are visualised in the scatterplots in Figure 3. Both pretraining and posttraining performance showed significant variation, with error rates ranging from 40 to 329 errors and from 32 to 189 before and after training, respectively. Across all groups, most students made fewer errors after training than before, with improvements of up to 50 errors being the most common. However, 12 students made more errors after training compared to their pretraining performance.

**Fig. 4:**
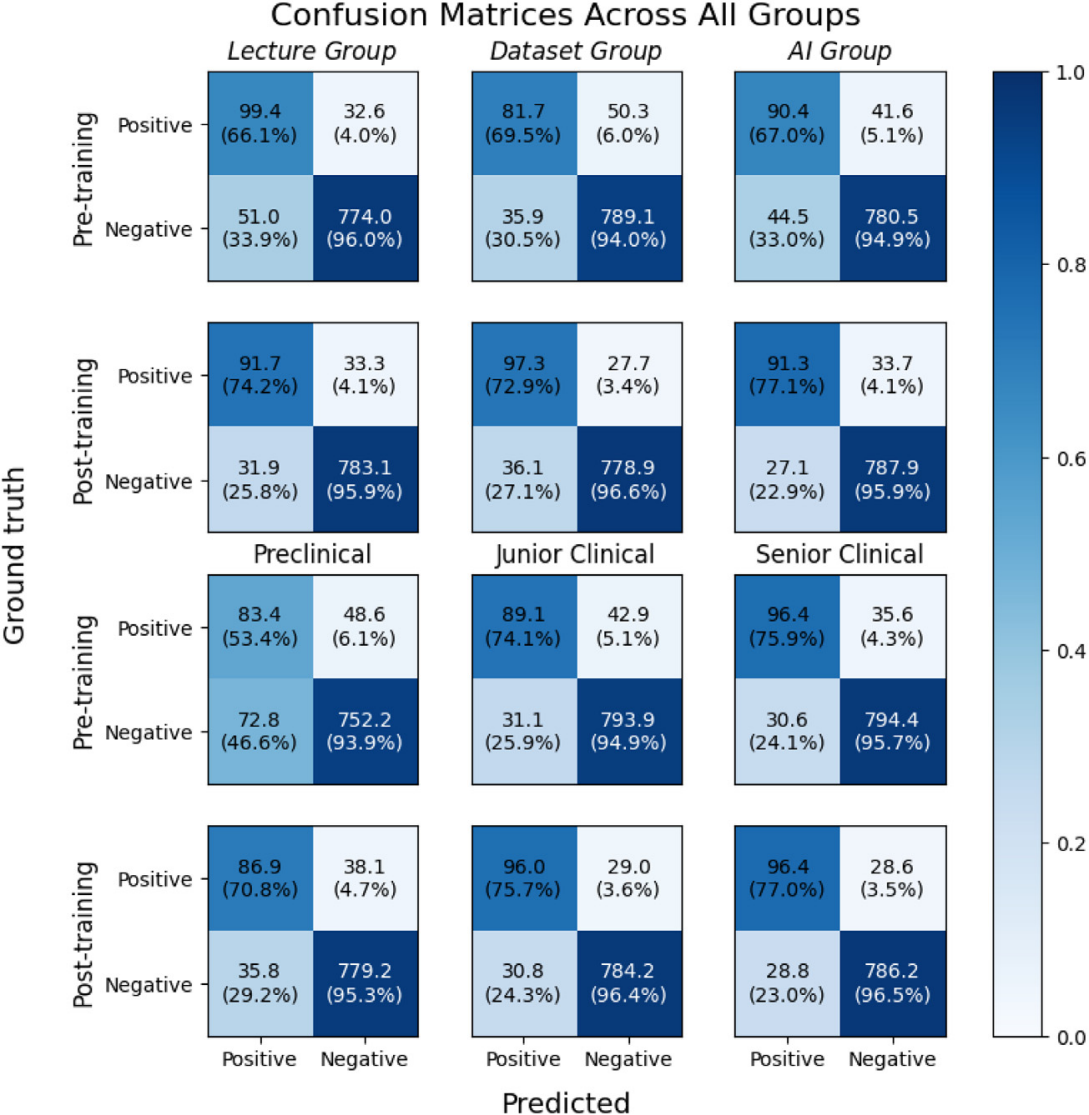
Confusion matrices with average values of true positive (TP), false negative (FN), false positive (FP), and true negative (TN) detections for each group before and after training. The presented values represent the means of all students in the respective subgroups. The colour scale and the percentages in parentheses indicate the relative proportion of mean TPs and FPs in the first column and the relative proportion of mean TNs and FNs in the second column for each subgroup.

Other diagnostic metrics also showed positive changes across all groups, as shown in Table 4, although many lacked statistical significance. The Dataset Group showed the greatest improvement in the primary outcome metric (sensitivity) and, according to the confusion matrices in Figure 4, was the only group that achieved a substantial increase in the average number of TPs. In addition to having the highest number of TPs, it also recorded the largest drop in FNs, indicating that this group was the most effective at detecting positive cases. However, its TNs slightly declined, resulting in a lack of improvement in specificity. The Lecture Group showed the largest improvement in TNs and the largest drop in FNs, leading to a significant rise in specificity. The AI Group was the only group that showed improvement across all detection categories (TP, FP, TN, and FN), resulting in the greatest improvements in precision and accuracy.

### Stage of academic study

**Fig. 5:**
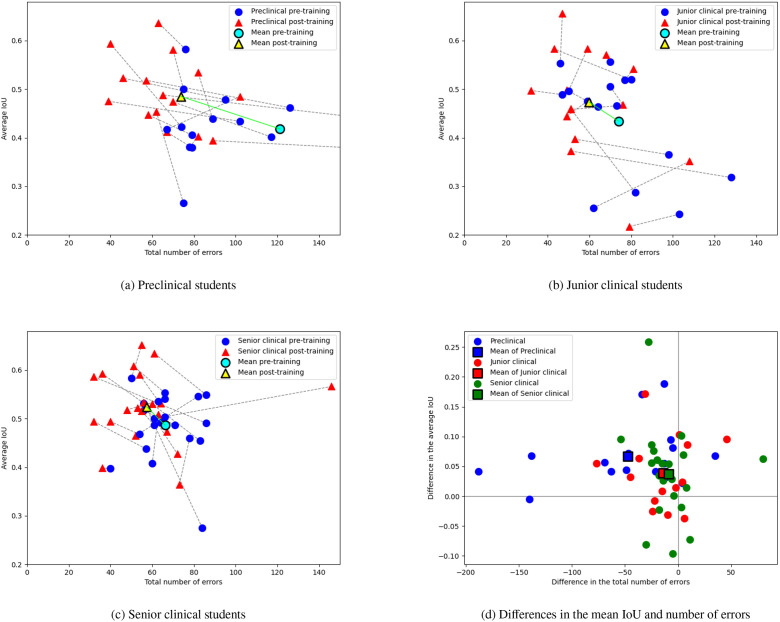
Mean IoU and total number of errors for preclinical (a), junior clinical (b), and senior clinical students (c) and their differences (d). Lines connect the pretraining and posttraining results of each student. An ideal result would be in the top left. For improved readability of the figure, the total number of errors displayed on the x-axis was capped at 150. Three outliers were affected by this cap, all in the group of preclinical students: 1 student improved from 329 to 189 errors, another improved from 203 to 65 errors, and the third student improved from 277 to 89 errors. IoU, intersection over union.

Table 5 and Figure 5 summarise scores in caries detection for preclinical, junior clinical, and senior clinical students. Preclinical students exhibited the highest number of errors both at baseline and after training. They also displayed the greatest IQR in posttraining error rates, indicating that the training effect was less predictable compared to more experienced students (see Table B.8 in Appendix B for the exact median values and IQR for each academic group). Junior clinical students showed a greater reduction in the number of errors compared to senior clinical students, although their improvement was less pronounced than that observed in preclinical students. The posttraining number of errors for junior clinical students was similar to that of senior clinical students, who did not exhibit a significant improvement after training.

**Table 5:** Pre- and posttraining results of preclinical students (first and second year), junior clinical students (third year), and senior clinical students (fourth and fifth year).

Characteristic	Errors	IoU	Sensitivity	Specificity	Accuracy	Precision	F1 score
Preclinical pretraining	121.3 ± 78.8	0.42 ± 0.07	0.63 ± 0.13	0.91 ± 0.11	0.87 ± 0.08	0.64 ± 0.21	0.60 ± 0.11
Preclinical posttraining	73.8 ± 35.1	0.48 ± 0.08	0.70 ± 0.11	0.96 ± 0.05	0.92 ± 0.04	0.75 ± 0.14	0.71 ± 0.09
*P* value	**.0012**	**<.0001**	.0715	**.0268**	**.0014**	**.0190**	**.0009**
Junior clinical pretraining	73.9 ± 22.4	0.43 ± 0.11	0.68 ± 0.16	0.96 ± 0.02	0.92 ± 0.02	0.76 ± 0.11	0.70 ± 0.11
Junior clinical posttraining	59.8 ± 19.3	0.47 ± 0.11	0.77 ± 0.11	0.96 ± 0.03	0.94 ± 0.02	0.78 ± 0.11	0.76 ± 0.07
*P* value	**.0362**	**.0085**	**.0016**	.5066	**.0480**	.2997	**.0122**
Senior clinical pretraining	66.2 ± 12.8	0.49 ± 0.07	0.73 ± 0.14	0.96 ± 0.02	0.93 ± 0.01	0.78 ± 0.09	0.74 ± 0.07
Senior clinical posttraining	57.4 ± 23.7	0.52 ± 0.07	0.77 ± 0.09	0.96 ± 0.03	0.94 ± 0.03	0.80 ± 0.12	0.77 ± 0.06
*P* value	.0686	**.0149**	.1115	.4348	.0982	.2507	**.0333**

Abbreviation: IoU, intersection over union.

Values are presented as mean ± standard deviation. The number of errors is the sum of false positives and false negatives. The bold *P* values indicate statistically significant improvement between pre- and posttraining results according to the permutation test (*P* < .05).

Moreover, preclinical students showed improvements across all diagnostic metrics. However, the increase in the primary outcome metric (sensitivity) only approached statistical significance (*P* = .0715). We speculate that this may be due to the limited sample size. In particular, they reduced their FPs by more than half, whereas the improvement observed among clinical students was minimal and nearly negligible. Junior clinical students exhibited a significant improvement in FNs and sensitivity without any adverse effect on specificity, which reflects their enhanced ability to detect TPs. Senior clinical students showed minimal improvement in detection, which was reflected in similarly small, if any, gains in accuracy, specificity, and precision.

## Discussion

This study compared 3 approaches for teaching caries detection in bitewing radiographs: a prerecorded online lecture, an annotated dataset of bitewings, and an AI-based web application that generated bounding boxes for automatic caries detection. To date, no study has directly compared AI-based training with other methods of education for caries detection. Existing research has primarily contrasted computer-aided training with no training at all.^34^, ^35^, ^36^

The number of errors was a critical metric in our study, highlighting both FPs and FNs. The average number of errors across 50 radiographs (1008 tooth surfaces) prior to training was 121.3 ± 78.8 for preclinical, 73.9 ± 22.4 for junior clinical, and 66.2 ± 12.8 for senior clinical students. After training with all methods, the average number of errors declined to 73.8 ± 35.1, 59.8 ± 19.3, and 57.4 ± 23.7 errors per 50 radiographs (993 tooth surfaces) for preclinical, junior clinical, and senior clinical students, respectively, bringing their performance closer to the error rate observed among dentists found in our previous study, which reported that dentists with varying levels of experience make 46.6 ± 22.1 errors on average when evaluated against the same standard.^3^ This highlights the students’ potential for improvement through targeted training. Mean IoU of matching annotations increased significantly in all student groups as well. However, values remained relatively low both before and after training, with the mean posttraining scores of academic groups ranging from 0.47 to 0.52. These modest differences are unlikely to be clinically meaningful, as the primary goal of radiograph interpretation training is the ability to detect caries rather than precisely determine its size at the pixel level, for which segmentation would be more suitable. Although IoU values around 0.5 may appear low, they are consistent with IoU found between dentists in our previous study (0.45-0.53 with an outlier of 0.36),^3^ as well as with findings of a recent systematic review, which reported even lower IoU values, especially in the detection of initial enamel lesions.^41^ This can be attributed to the blurred lesion boundaries on bitewings and the fact that bounding boxes include not only pixels of carious lesions but also sound tissues and background.

The effect of training varied notably. The group that trained with the prerecorded lecture achieved the largest reduction in FPs, likely because the lecture was the only method that directly addressed common diagnostic errors (eg, the burnout effect, enamel overlap, and nonhomogeneous or radiolucent restorations). This could help students avoid such errors, leading to a reduction in FP annotations. In contrast, training with the annotated dataset led to the largest reduction in FNs and the largest rise in TPs, which is consistent with the performance of the dataset’s annotator, who showed a sensitivity of 88%, with only 16 FNs per 50 images. The annotator’s 116 TPs (20 more than the mean among senior clinical students) informed students of lesions they had missed during the annotation of the initial dataset. As a result of the substantial increase in TPs and corresponding reduction in FNs, this group exhibited the largest gain in sensitivity. However, specificity did not improve, which may reflect the influence of the dataset annotator, whose baseline specificity was only marginally higher than that of the students prior to training. We used a single annotator for training, as this setup was intended to simulate a teaching scenario with interaction guided by 1 young instructor.

Students using the AI-generated annotations improved across all predictions (TPs, FPs, TNs, and FNs). Moreover, students using the AI application exhibited the largest decrease in the mean number of errors following training; however, their median improvement was smaller compared to the other groups. This pattern suggests that a few individuals made substantial gains, while overall progress was less uniform across the group. We speculate that this may be due to the training being entirely self-directed. Students could engage with the application as much or as little as they wished; some may have uploaded numerous images beyond the pre-uploaded dataset and spent considerable time exploring its features, while others might have used it only a few times before disengaging. Nevertheless, the interactivity of the application may have contributed to students’ adherence, with all participants completing the training using the AI-generated bounding boxes. In contrast, the other teaching methods were less interactive. The lecture consisted primarily of didactic instruction, and the annotated dataset offered a passive review of examples. In these less engaging training methods, 2 students in each group discontinued participation, despite them being less time-consuming than the AI-based training. The varying impact of AI for each dentist was reported by a randomised trial by Mertens et al,^25^ which highlights the importance of reflecting on its user-specific benefits and risks. However, the improvement in error rate with the AI application was not significantly different from that of the remaining 2 groups in the Kruskal-Wallis test.

To facilitate comparison with other studies, we evaluated additional metrics recommended for dental computer vision studies.^40^ Sensitivity was prespecified as the primary outcome metric, while specificity, accuracy, precision, and F1 score were treated as secondary, exploratory metrics. Although median values and interquartile ranges were reported because of the nonnormal data distribution, mean values are discussed to enable direct comparisons with previously published studies. However, direct comparison between studies remains challenging because many studies do not specify the criteria used to determine when annotations are considered matching. In our study, we defined a match as occurring when the centroid of 1 annotation lay within the other or vice versa, while other studies may use different criteria, such as an IoU threshold. Overall, students showed improvement after training, but significant differences between groups were only reported in the improvement of sensitivity and F1 scores. Specifically, students who trained with the annotated dataset outperformed those who trained with the prerecorded lecture.

This study observed high baseline sensitivity levels, even for students with limited training: 63% for preclinical, 68% for junior clinical, and 73% for senior clinical students. Comparable studies reported a sensitivity of 48% to 52% for students in the second^34^ and third^35^^,^^36^ academic years. The higher sensitivity scores in this study may have several reasons. First, unlike comparable studies, this study included the minority set of tentative annotations, which consists of lesions marked by a single expert. These are considered diagnostically uncertain, rather than being TP or TN. They mostly include incipient carious lesions, which are hardest to detect and most often missed, thereby lowering sensitivity. This is supported by findings from a systematic review, which showed that sensitivity increases when initial lesions are excluded from analysis, reaching over 60% for cavitated proximal lesions.^2^ A previous study that compared dentists with the same reference standard found the average sensitivity of both novices and expert dentists to be above 90%, further highlighting the effect of excluding the diagnostically uncertain annotations.^3^ In contrast, the studies by Devlin et al^35^ and Schropp et al^36^ excluded dentin lesions, thereby limiting the analysis to enamel lesions. Second, the difference in baseline sensitivity may be attributed to varying levels of knowledge among students and dentists, which are influenced by their education and experience at different institutions.

In the group trained with the AI application, sensitivity increased from 68% to 73%, while the F1 score rose from 68% to 75%. These results closely align with the posttraining sensitivity of 76% and F1 score of 74% reported by Ayan et al,^34^ who, similarly to our approach, used the AI application exclusively during the training phase. In contrast, Devlin et al^35^ allowed students to use the AI application directly during testing, which naturally led to a larger performance gap between students with and without AI assistance, with sensitivity increasing from 50% to 80%. Additionally, the sensitivity of dentists and faculty members using an AI tool for caries detection in radiographs has been reported to range from 76% to 96%.^24^^,^^25^^,^^42^ The higher sensitivity range with the use of AI may be attributed not only to the clinicians’ level of experience but also to the quality of the AI system used.

Specificity, which reflects the ability to correctly identify negative cases, improved significantly only in the group trained with the prerecorded lecture addressing potential errors, rising from 94% to 96%. In contrast, the AI-trained students did not show a statistically significant increase, although their posttraining specificity was comparable at 97%. Findings from other studies reveal mixed results. While Ayan et al^34^ reported significant improvements in both sensitivity and specificity, Schropp et al^36^ found results more similar to ours: specificity remained unchanged in the AI group (86%) but increased significantly from 80% to 89% in the group that received no additional training beyond annotating the initial dataset. This may reflect a common trade-off, as AI systems tend to boost sensitivity by flagging more potential positives, which can also increase FPs and hence decrease specificity. Accuracy improved significantly across all groups in our study. The baseline value was 91% for each group, increasing to 94% with AI-assisted training and 93% with other e-learning methods. In comparison, Ayan et al^34^ reported a baseline accuracy of 84%, which rose markedly to 89% with AI-assisted training and to 86% without AI. Since accuracy is influenced by disease prevalence, differences in baseline values may be partly explained by the lower prevalence of caries in our study (12.6%-18.2%) compared with that reported by Ayan et al^34^ (25.6%). This lower prevalence likely contributed to the relatively high baseline accuracy observed in our dataset. However, the Kruskal-Wallis test did not reveal any significant differences in specificity or accuracy between groups in our study.

The second aim of our study was to evaluate whether students’ performance in caries detection is influenced by the stage of their studies. A trend of greater improvement for less experienced students was observed, with preclinical students exhibiting the most substantial gains after training. However, pairwise comparisons showed no significant differences in improvement between students grouped by their stage of academic study. Moreover, while most students demonstrated improvement after training, some performed worse posttraining compared to their baseline performance. This suggests that the effect of training may vary across individuals. Notably, the largest range of improvement in the number of errors was observed in the preclinical group, indicating that their response to training was more heterogeneous compared to the other groups.

This study has several limitations that should be acknowledged. First, the diversity of opinions in clinical practice was reflected by the assignment of annotations to the majority and minority reference standards. This approach was incorporated to ensure an unbiased evaluation, which reflects the low interobserver agreement reported by several studies.^2^^,^^3^ Comparable studies commonly determine the reference standard through majority voting,^16^^,^^18^^,^^22^^,^^24^^,^^25^^,^^34^, ^35^, ^36^^,^^42^ which neglects the minority opinion and was reported to be suboptimal.^43^ While histologic validation would provide a solid reference, it is not feasible for large-scale studies of caries detection due to its invasive nature. Srivastava et al^23^ attempted to solve this issue by employing clinical visual-tactile examination to verify caries diagnosis, but this approach is not optimal either, as the clinical examination has a lower sensitivity in proximal caries detection compared to radiographic analysis, reaching only 32% for dentin lesions.^44^ Second, while the combined results showed statistically significant improvements after training, the differences between groups were not always significant, which may indicate insufficient statistical power. The sample size was determined by feasibility, as the study employed a convenience sampling approach and included all students who were willing to participate. Furthermore, the inclusion of an additional control group receiving no training would have strengthened the study design, but there were not enough participants to include an additional group.

Considering the limitations, future research with a larger number of participants would help strengthen the findings. The generalisability of the results could be further improved by recruiting students from multiple institutions to better reflect diverse educational backgrounds, expanding the bitewing dataset to include images from additional sources, and implementing multiple AI-based applications for their training. Future studies could also explore how different educational approaches influence the detection of caries at various stages of lesion severity, as well as evaluate student engagement and preferences across both AI and non-AI training methods in dentistry.

## Conclusion

Taking the limitations of this study into account, it can be concluded that all training methods led to improvements in caries detection. The AI application produced improvements similar to the established e-learning methods, but training with the AI-generated annotations led to a higher variability in outcomes, which may reflect differences in how each student chose to engage with the application. These findings support the need for further investigation into the individual effects of Al-based tools on the performance of dental students and dentists. When comparing the stage of study, no significant difference was found between academic groups, but the largest reduction in error rate was observed among preclinical students with minimal prior experience. In contrast, the most experienced students, whose error rates after training approached that of dentists, did not exhibit significant improvements.

## Declaration of generative AI and AI-assisted technologies in the manuscript preparation process

During the preparation of this work, the authors used ChatGPT (GPT-5, OpenAI) to optimise phrasing and ensure clarity. After using this tool, the authors reviewed and edited the content as needed and took full responsibility for the content of the published article.

## Author contributions

Valeria Nagyová: Writing—original draft, Investigation, Methodology, Project administration. Dominik Blaňár: Writing—original draft, Data curation, Formal analysis, Methodology, Software, Validation, and Visualisation. Jan Kybic: Writing—review & editing, Methodology, Funding acquisition, Supervision. Falk Schwendicke: Writing—review & editing, Validation. Antonín Tichý: Writing—review & editing, Funding acquisition, Supervision, Resources, Conceptualisation.

## Funding

This work was supported by the General University Hospital in Prague (grant GIP-21-SL-01-232); Charles University, Cooperatio Dental Medicine/LFl (grant 207030); and the Ministry of Education, Youth and Sports of the Czech Republic, Operational Programme Research, Development and Education (OP VVV) (grant CZ.02.1.01/0.0/0.0/16_019/0000765).

## Ethics approval

This research was approved by the Ethics Committee of the General University Hospital in Prague (protocol number 64/24), and all procedures were performed in compliance with relevant laws and institutional guidelines.

## Declaration of competing interests

The authors declare the following financial interests/personal relationships which may be considered as potential competing interests: Given his role as an editor of the Artificial Intelligence in Dentistry special issue of the *International Dental Journal*, Antonín Tichý, DMD, PhD, had no involvement in the peer review of this article and had no access to information regarding its peer review. Full responsibility for the editorial process for this article was delegated to another journal editor. Other authors declare that they have no known competing financial interests or personal relationships that could have appeared to influence the work reported in this paper.

## Appendix Supplementary materials

Image, application 1

## Footnotes

Supplementary material associated with this article can be found in the online version at doi:10.1016/j.identj.2026.109567.
