# Supplementary material for: Student Performance in Bitewing Caries Detection: Artificial Intelligence Versus Alternative E-learning
Source: Int Dent J. 2026 Apr 30;76(4):109567. doi: 10.1016/j.identj.2026.109567 (PMC13144576; doi:10.1016/j.identj.2026.109567)
Supplement: Supplementary file 1 [file mmc1.pdf]

Appendix A. Comparison with the reference standard

For each annotator $a \in \mathrm{A}$ and each image $i \in D$, a set of bounding boxes B_{ia}, denoted as B, was compared with set of annotations $\mathrm{B}'_i \in \mathrm{S}$, denoted as B', for the given image. Two bounding boxes $b \in \mathrm{B}$ and $b' \in \mathrm{B}'$ were considered to match, if the centroid of one box lied inside the other, or vice versa. This condition is expressed as follows:

$$b \cong b' \iff \mathrm{centroid}(b) \in b' \lor \mathrm{centroid}(b') \in b \tag{A.1}$$

Sets of correct annotations Ω and a set of tentative annotations Ω' were created using a simple greedy algorithm similar to that used in the previous project[3], where sets of annotations used for comparison were $\mathrm{B}' \in \mathrm{S}$ and $\tilde{\mathrm{B}} \in \mathrm{S}'$. The algorithm is as follows:

1. Find the largest box b from B.
2. Find a corresponding box $b' \in \mathrm{B}'$ such that $b' \cong b$ (see (A.1)), i.e., the boxes match. If there are multiple such b', choose the one that maximizes the IoU $\frac{|b \cap b'|}{|b \cup b'|}$. If it is not unique, pick the largest b'.
3. If a match b' was found, insert (b, b') into Ω and remove b from B and b' from B'. If no match b' was found from B', find a corresponding box $\tilde{b} \in \tilde{\mathrm{B}}$ as in step 2. If such a match $\tilde{b}$ was found, insert $(b, \tilde{b})$ into Ω' and remove b from B and $\tilde{b}$ from $\tilde{\mathrm{B}}$.
4. Repeat until B is empty or all boxes have been considered.

Then, the number of TP annotations for the current image i and the current annotator a is the number of matched annotations in Ω. The number of FP annotations for the current image i and the current annotator a is the number of unmatched annotations in B. The number of FN annotations for the current image i and the current annotator a is the number of unmatched annotations in B'. The number of TN annotations for an annotator a across the entire dataset was calculated using the total number of tooth surfaces N for a given dataset D, and the formula is as follows:

$$\mathrm{TN}_a = N - |\mathrm{S}'_D| - \sum_{i \in D} (\mathrm{TP}^i_a + \mathrm{FP}^i_a + \mathrm{FN}^i_a) \tag{A.2}$$

The number of errors for the current image i and the current annotator a is the sum of the FP and FN annotations or the number of remaining unmatched boxes in sets B and B'.

$$e^i_a = \mathrm{FP}^i_a + \mathrm{FN}^i_a = |\mathrm{B}| + |\mathrm{B}'| \tag{A.3}$$

The total number of errors per annotator a is the sum over all images.

The mean IoU was calculated for each pair of matching boxes $(b, b') \in \Omega$ per image, and the mean value was calculated per annotator a over all images.

$$\mathrm{IoU}_a = \frac{1}{|D|} \sum_{i \in D} \frac{\sum_{(b,b') \in \Omega^i_a} \mathrm{IoU}(b, b')}{|\Omega^i_a|} \tag{A.4}$$

Appendix B. Median and interquartile values

Table B.6: Pre- and post-training results of all 52 students: median and interquartile range $[Q1 - Q3]$.

Median $[Q1 - Q3]$	**Errors**	**IoU**	**Sensitivity**	**Specificity**	**Accuracy**	**Precision**	**F1 score**
**All students pre-training**	74.5 [61.8 - 84.5]	0.47 [0.40 - 0.51]	0.71 [0.60 - 0.79]	0.96 [0.94 - 0.98]	0.92 [0.91 - 0.94]	0.75 [0.68 - 0.84]	0.72 [0.65 - 0.76]
**All students post-training**	57.5 [49.0 - 70.0]	0.50 [0.45 - 0.57]	0.77 [0.69 - 0.83]	0.97 [0.95 - 0.98]	0.94 [0.93 - 0.95]	0.79 [0.71 - 0.88]	0.76 [0.72 - 0.80]
	$\boldsymbol{p<0.0001}$	$\boldsymbol{p<0.0001}$	$\boldsymbol{p=0.0011}$	$\boldsymbol{p=0.0411}$	$\boldsymbol{p=0.0001}$	$\boldsymbol{p=0.0186}$	$\boldsymbol{p<0.0001}$

Note: Q1 represents the first quartile (25th percentile) and Q3 represents the third quartile (75th percentile). The bold p-values indicate statistically significant improvement between pre- and post-training results according to the permutation test ($p < 0.05$). The number of errors is the sum of false positives (FP) and false negatives (FN). IoU stands for intersection over union.

Table B.7: Pre- and post-training results of the *Lecture Group*, the *Dataset Group* and the *AI Group*: median and interquartile range [$Q1 - Q3$].

Median [$Q1 - Q3$]	**Errors**	**IoU**	**Sensitivity**	**Specificity**	**Accuracy**	**Precision**	**F1 score**
***Lecture Group* pre-training**	68.5 [54.5 – 76.3]	0.50 [0.45 – 0.54]	0.78 [0.65 – 0.84]	0.96 [0.94 – 0.98]	0.93 [0.92 – 0.94]	0.75 [0.68 – 0.85]	0.75 [0.71 – 0.79]
***Lecture Group* post-training**	56 [50.5 – 64.8]	0.56 [0.45 – 0.60]	0.75 [0.67 – 0.82]	0.97 [0.96 – 0.99]	0.94 [0.93 – 0.95]	0.81 [0.74 – 0.90]	0.76 [0.72 – 0.79]
	***p*=0.0049**	***p*=0.0003**	*p*=0.7309	***p*=0.0226**	***p*=0.0092**	***p*=0.0321**	*p*=0.1358
***Dataset Group* pre-training**	80.0 [66.0 – 86.0]	0.45 [0.41 – 0.49]	0.61 [0.50 – 0.74]	0.97 [0.94 – 0.98]	0.92 [0.91 – 0.93]	0.75 [0.69 – 0.84]	0.69 [0.59 – 0.71]
***Dataset Group* post-training**	56.0 [49.0 – 67.0]	0.51 [0.46 – 0.53]	0.79 [0.75 – 0.84]	0.96 [0.95 – 0.98]	0.94 [0.93 – 0.95]	0.78 [0.70 – 0.85]	0.78 [0.72 – 0.80]
	***p*=0.0302**	***p*=0.0006**	***p*=0.0001**	*p*=0.5167	***p*=0.0381**	*p*=0.3757	***p*=0.0008**
***AI Group* pre-training**	74.0 [61.5 – 92.0]	0.46 [0.40 – 0.50]	0.71 [0.63 – 0.77]	0.96 [0.94 – 0.98]	0.92 [0.90 – 0.94]	0.77 [0.66 – 0.81]	0.72 [0.66 – 0.76]
***AI Group* post-training**	61.0 [46.0 – 71.5]	0.47 [0.42 – 0.51]	0.76 [0.66 – 0.82]	0.97 [0.95 – 0.99]	0.94 [0.92 – 0.95]	0.81 [0.70 – 0.88]	0.75 [0.72 – 0.80]
	***p*=0.0037**	***p*=0.0481**	***p*=0.0450**	*p*=0.0647	***p*=0.0053**	***p*=0.0314**	***p*=0.0032**

Note: Q1 represents the first quartile (25th percentile) and Q3 represents the third quartile (75th percentile). The bold *p*-values indicate statistically significant improvement between pre- and post-training results according to the permutation test ($p < 0.05$). The number of errors is the sum of false positives (FP) and false negatives (FN). IoU stands for intersection over union.

Table B.8: Pre- and post-training results of preclinical students (first and second year), junior clinical students (third year) and senior clinical students (fourth and fifth year): median and interquartile range [$Q1 - Q3$].

Median [$Q1 - Q3$]	**Errors**	**IoU**	**Sensitivity**	**Specificity**	**Accuracy**	**Precision**	**F1 score**
**Preclinical pre-training**	84.0 [75.8 – 119.3]	0.42 [0.38 – 0.44]	0.64 [0.54 – 0.73]	0.95 [0.92 – 0.98]	0.91 [0.88 – 0.92]	0.69 [0.55 – 0.77]	0.62 [0.56 – 0.69]
**Preclinical post-training**	66.0 [57.8 – 82.0]	0.48 [0.44 – 0.53]	0.70 [0.61 – 0.76]	0.97 [0.96 – 0.98]	0.93 [0.91 – 0.94]	0.78 [0.67 – 0.84]	0.69 [0.68 – 0.76]
	***p*=0.0012**	***p*<0.0001**	*p*=0.0715	***p*=0.0268**	***p*=0.0014**	***p*=0.0190**	***p*=0.0009**
**Junior clinical pre-training**	70.0 [60.5 – 81.0]	0.48 [0.34 – 0.51]	0.67 [0.60 – 0.79]	0.97 [0.95 – 0.98]	0.93 [0.92 – 0.94]	0.77 [0.70 – 0.84]	0.73 [0.67 – 0.75]
**Junior clinical post-training**	51.0 [49.0 – 72.0]	0.47 [0.42 – 0.56]	0.80 [0.74 – 0.83]	0.97 [0.95 – 0.99]	0.95 & 0.03[0.92 – 0.95]	0.79 [0.71 – 0.86]	0.78 [0.75 – 0.80]
	***p*=0.0362**	***p*=0.0085**	***p*=0.0016**	*p*=0.5066	***p*=0.0480**	*p*=0.2997	***p*=0.0122**
**Senior clinical pre-training**	64.0 [57.0 – 78.0]	0.49 [0.46 – 0.54]	0.77 [0.65 – 0.82]	0.96 [0.95 – 0.98]	0.93 [0.92 – 0.94]	0.77 [0.73 – 0.85]	0.76 [0.71 – 0.78]
**Senior clinical post-training**	55.0 [48.0 – 63.0]	0.52 [0.49 – 0.59]	0.78 [0.75 – 0.85]	0.98 [0.95 – 0.99]	0.94 [0.93 – 0.95]	0.80 [0.72 – 0.88]	0.77 [0.74 – 0.81]
	p=0.0686	***p*=0.0149**	*p*=0.1115	*p*=0.4348	*p*=0.0982	*p*=0.2507	***p*=0.0333**

Note: Q1 represents the first quartile (25th percentile) and Q3 represents the third quartile (75th percentile). The bold *p*-values indicate statistically significant improvement between pre- and post-training results according to the permutation test ($p < 0.05$). The number of errors is the sum of false positives (FP) and false negatives (FN). IoU stands for intersection over union.
